# Using Activity-Related Behavioural Features towards More Effective Automatic Stress Detection

**DOI:** 10.1371/journal.pone.0043571

**Published:** 2012-09-19

**Authors:** Dimitris Giakoumis, Anastasios Drosou, Pietro Cipresso, Dimitrios Tzovaras, George Hassapis, Andrea Gaggioli, Giuseppe Riva

**Affiliations:** 1 Informatics and Telematics Institute, Centre for Research and Technology Hellas, Thermi, Thessaloniki, Greece; 2 Department of Electrical and Computer Engineering, Aristotle University of Thessaloniki, Thessaloniki, Greece; 3 Department of Electrical and Electronic Engineering, Imperial College London, London, United Kingdom; 4 Applied Technology for Neuro-Psychology Lab, IRCCS Istituto Auxologico Italiano, Milan, Italy; 5 Psychology Department, Catholic University of Milan, Milan, Italy; Ecole Normale Supérieure, France

## Abstract

This paper introduces activity-related behavioural features that can be automatically extracted from a computer system, with the aim to increase the effectiveness of automatic stress detection. The proposed features are based on processing of appropriate video and accelerometer recordings taken from the monitored subjects. For the purposes of the present study, an experiment was conducted that utilized a stress-induction protocol based on the stroop colour word test. Video, accelerometer and biosignal (Electrocardiogram and Galvanic Skin Response) recordings were collected from nineteen participants. Then, an explorative study was conducted by following a methodology mainly based on spatiotemporal descriptors (Motion History Images) that are extracted from video sequences. A large set of activity-related behavioural features, potentially useful for automatic stress detection, were proposed and examined. Experimental evaluation showed that several of these behavioural features significantly correlate to self-reported stress. Moreover, it was found that the use of the proposed features can significantly enhance the performance of typical automatic stress detection systems, commonly based on biosignal processing.

## Introduction

Growing interest has surrounded the roles of technology in emotions, and in particular, in psychological stress. As such, automatic stress detection has become a challenging issue, for both research and the clinical practice. At the moment, physiological measurements and self-report questionnaires are the most common methods used to automatically detect stress [1], [2]. Although questionnaires are affected by personal convictions [3] and biosensors are often too obtrusive [4], last decades' research has demonstrated an increasing quality of these methods and related technologies. In this line, effort has been made to improve the performance, and also to reduce the obtrusiveness of the adopted systems. However it still remains partially unclear how stress can be effectively detected with the help of systems that retain an increased degree of unobtrusiveness.

According to Cohen, Janicki-Deverts, and Miller [5], psychological stress occurs when an individual perceives that the environmental demands exceed his or her adaptive ability to meet them. This gap gives rise to the labeling of oneself as stressed and elicits a concomitant negative emotional response. In physiological measures, such a response can lead to increased stress hormone levels, blood pressure [6], heart rate, pupil dilation, and skin conductivity [7], [8]. In activity-related behavior such an emotional response can lead to a wide range of “behavioural symptoms”; for example, hands and foot trembling [9], body hyperactivity [2], [10], compulsive movement [11], and faster eye gaze [12].

Over the last few decades, many factors, such as the lower technology price and its higher availability, portability, and usability, have allowed a closer connection and interaction between automatic detection systems and affective models. A vivid example of such joining is the promising field of Affective Computing [13], [14], which has among others, the aim to identify emotions during human-computer interaction, by examining biological signals [15], [16], facial expressions [17], speech [18], hands [19], and further parameters.

Several studies have shown interesting results that support the feasibility of detecting affective states through psychophysiological data acquisition and analysis [20]. For example, the affective computing group at MIT, led by Rosalind W. Picard (the pioneer in the field who also coined the term), conducted several research studies that highlighted the use of psychophysiological measures towards deducing and classifying emotional states. In particular, researchers have highlighted the usefulness of wearable biosensors that detect changes in physiological and subsequently, affective states [7], [14], [15].

However, considering the practical applicability of such methodologies, a major problem with wearable biosensors is related to obtrusiveness. In fact, the regularly utilized biosensors are not transparent to subjects, something that may even affect the study goals, i.e. in studying stress, there can be cases where subjects become stressed as a consequence of the biosensors themselves [4]. Nevertheless, until now, the common tactic has been to decrease the obtrusiveness of such devices, sometimes also making them almost invisible, i.e. by using an electrocardiogram under a t-shirt that transmits data to a smart phone [4] or a skin conductance recorder to wear on the wrist [21].

In an effort to augment automatic affect detection with less obtrusive monitoring methods, the applicability of automatically extracted activity-related parameters has been recently examined [22]. Activity-related behaviours suggest a clear aspect of continuous regulatory actions, observable in movement qualities, contours, expressions, and also perceived in vocal tonality [23]. In this respect, the body attitude is related to the constant shape of the body, its general pose and the distinct location of its parts [24], [25]. This concept recalls the so called “background emotion” [23] or “state of mind” that considers how one perceives oneself and how this affects others.

In a more general view, gesture and posture are considered as parts of a wider semiotic system that underlies human communication. Along this line, it has been reported that attitude, intention, and, in general, meaning, are expressed only in part by verbal content, and as much or even more so through nonverbal channels [17], [23], [26], [27], [28]. In this perspective, nonverbal behaviours could be interpreted to indicate the “unsaid” elements representing our internal states.

Human gestural parameters have recently been extracted from monitoring video sequences, and it was shown that they have interesting potential towards automatic affect recognition [22]. The latter work drew inspiration from [29], where the degree of dependence between body movements and postures and certain emotions, like joy, happiness, anger, etc had been investigated. However, it has to be noted that, to our knowledge, no study has explored until now the potential of activity-related behavioral features, in respect to the practical problem of automatic stress detection. These features could be collected at distance through the use of appropriate sensors or cameras, being this way totally unobtrusive. This consideration needs to be reviewed in further studies, in order to understand the implications of such features for the research and for the health care practice. Following this line, a study aiming to automatically detect the stress level of participants has been conducted herein. Psychological self-reports and common physiological measures were used, and the latter were compared to a less obtrusive technology, mainly based on a low cost, single view depth imaging camera (Microsoft Kinect [30]). In the context of our study, the following research questions are put forth:

**RQ1:** Is there a relationship between behavioural features that can be automatically extracted from a computer system, and the self-reported stress levels of subjects?

**RQ2:** Is it possible to enhance stress detection effectiveness by adding automatically detected behavioural information to standard systems?

The present work tries to answer both these research questions by comparing standard psychological questionnaires, common psychophysiological measures, and activity-related behavioural indexes. An experiment was deployed with the aim to collect appropriate video and accelerometer data from participants who followed a stress induction protocol. Galvanic Skin Response (GSR) and Electrocardiogram (ECG) biosignals were also recorded during the experiment. An explorative study was then conducted, to identify whether behavioural features extracted from video and accelerometer recordings can improve the effectiveness of automatic stress detection. At this purpose, a large set of behavioural features were extracted from the collected data, and their relation to self-reported stress was examined. Many mixed linear hierarchical regressions were computed, to take into consideration the nested structure of the data. Moreover, utilizing a linear classifier, it was found that the proposed behavioural features were capable to significantly enhance effectiveness of automatic stress detection, compared to the results obtained when only common physiological features, extracted from the monitored biosignals, were used.

## Materials and Methods

In order to collect appropriate data, a stress-induction experiment was conducted, as explained in the following.

### Participants

Twenty one right-handed subjects (4 women, 17 men, Mean = 30.4 years, SD = 3.7) participated in the experiment, which was conducted in the premises of Informatics and Telematics Institute, Centre for Research and Technology Hellas (CERTH-ITI) in Thessaloniki, Greece. All subjects gave written informed consent to the experimental procedure, which was approved by the local ethics committee of Centre for Research and Technology Hellas.

### Hardware Setup

**Figure 1:**
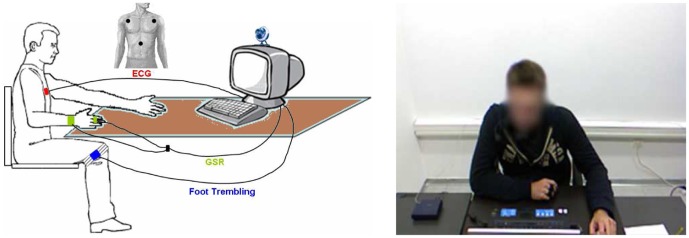
Illustration of the sensors attached on a Subject (a) and screenshot (from Kinect camera) of the Subject during the experiment (b).

Video data was collected through a Microsoft Kinect [30] camera placed opposite to the participant, at a distance of around 2 1/2 meters. Accelerometer data was collected from two tri-axial accelerometer sensors developed by Phidget Corp. [31] that were placed at the participant's knees, with the aim to detect foot trembling. Moreover, physiological (GSR and ECG) data was collected using a Procomp5 [32] Infinity device. For GSR signal acquisition, one two-electrode GSR sensor was placed at the subject's index and middle fingers of the non-dominant hand. Also, one three-electrode ECG sensor was placed at the subject's chest, covering Eindhoven's triangle. The overall sensor setup of the study and a sample screenshot taken from the video recordings are shown in Figure 1.a and Figure 1.b, respectively.

### Stimuli and Procedure

#### Stimuli

The stress-induction stimuli of the experiment was based on a custom stroop colour word test [33] application (Figure 1). The stroop test has been commonly utilized in the past so as to examine attention and cognitive flexibility [34], emotion perception [35], as well as the effect of stress manipulation on cognitive performance [36]. However, due to the fact that it is as a mental task whose difficulty may substantially increase (through manipulation of task pacing etc), it has also been considered during the recent years as capable to form the basis for stress-induction stimuli [20], [37], [38]. Following this line, the stroop test was used in our work, so as to provide a mental task of increased difficulty, within a stressor framework that was also based on time pressure. Eventually, the stressor of our experiment was mainly based on two parameters, i.e. time pressure and increased task difficulty, commonly known as capable to induce stress [39], [40]. As explained in the following, these two factors were manipulated throughout the different conditions of our experiment, during the participants' interaction with our custom Stroop-based application.

In our specific stroop test, five colours were utilized, namely red, green, blue, yellow and pink. Two versions of the test were implemented: In Version A, the subject was presented with five buttons labelled after the specific colours. For each question, the subject had to press the correct button. In Version B, speech recognition was utilized; the subject had to speak out the name of the correct colour.

#### Experimental Protocol

During the experiment, the subject was initially briefed and asked to sign the consent form. Then, the sensors were installed. All experiments lasted for about one hour in total, including the sensors setup phase. The stress induction aim of the experiment was kept hidden from participants throughout its duration. This way, it was ensured that stress induction would occur naturally through the stressor and self reports would have not been biased from subjects' prior knowledge over the fact that they “should get stressed”. Moreover, subjects were unaware of the processing methods that would have subsequently been applied over their video and accelerometer recordings. It was thus also ensured, that collected data would have not been biased from manipulations of subjects that might have been aware of their body language.

The experimental procedure consisted of the following eight conditions:

*Rest:* The subject was asked to relax for two minutes with eyes closed. GSR and ECG baseline data were recorded during this period.

*Condition 0:* The subject watched a relaxing video, compiled from pictures of Greek islands. GSR, ECG, video and accelerometer data were recorded during this period, which lasted for one and a half minutes. Data from the same sensors/devices was also recorded during the rest of the conditions (1–6). At the end of condition 0, the subject answered the post-condition questionnaire, which is explained below.

*Condition 1:* The subject played an easy (congruent) version of the Stroop colour word test (Version A), where the font colour was always same as the colour name displayed. The time limit for each question was five seconds. The condition ended when sixty questions had been answered, and then, the subject filled in the post-condition questionnaire.

*Condition 2:* The same specifics as in condition 1 were followed, only this time, Version B of the stroop test was used, and thus the subject had to speak out the font colour instead of clicking the colour buttons.

**Figure 2:**
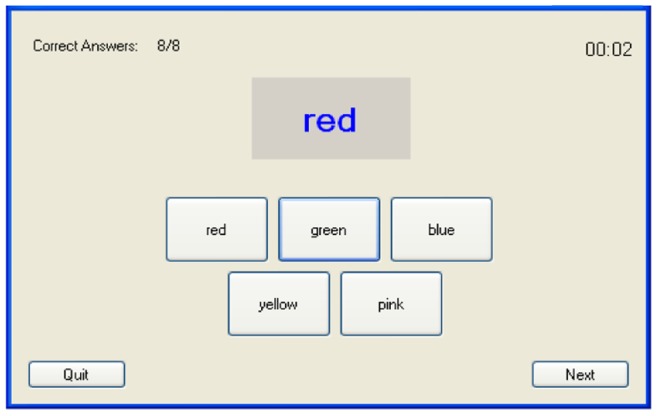
Screenshot of the experiment stimuli.

*Condition 3:* The subject played the typical Stroop colour word test (Version A), where the font colour was always different than the colour name displayed (Figure 2). The time limit for each question was three seconds. After two minutes of playing, the game automatically paused for one minute and automatically resumed, so as for the subject to play for another two minutes. At the end, the subject completed the post-condition questionnaire.

*Condition 4:* The same specifics as in condition 3 were followed, only this time, Version B of the Stroop test was used.

*Condition 5:* The same specifics as in condition 3 were followed, however, this time the subject played a Stroop colour word test of increased difficulty (Version A); the font colour was always different than the colour name displayed, and also after each of the subject's answer, the order of buttons changed in a random manner. The time limit for each question was two seconds.

*Condition 6:* The same specifics as in condition 5 were followed, however this time, the subject played a Stroop test of increased difficulty (Version B), where three colour words were presented in each question instead of the typical one. Two out of the three words had always the same colour. The subject had to identify the dominant font colour and speak it out loud.

Summarizing, the experiment consisted of eight different conditions, one for recording of baseline biosignals data (as typically done in biosignals-based affect detection studies, e.g. [37], [41], [42]) and seven (condition 0–condition 6) for collecting both physiological and behavioural measurements during periods with presence and absence of stress.

From each participant, we needed as many recordings of subjects as possible, taken both during not-stressed and stressed states. For this purpose, the initial, Rest condition, was followed from condition 0, from which physiological and behavioural measurements were to be collected, during a period with potentially complete absence of stress. Then, the interaction with the Stroop-based application involved three different levels of difficulty; a very easy level (conditions 1 and 2), a moderately difficult level (conditions 3 and 4) and a very difficult one (conditions 5 and 6). Whereas the first two levels had a strong resemblance to (congruent-incongruent) stroop tests that have been used in the past [37], our third level involved a stroop test variation of very high task difficulty and time pressure. As such, conditions 5 and 6 were expected to prove particularly stressful.

Each of the above difficulty levels consisted of two conditions, one of which employed the version of our Stroop-based application with button-press (Version A, used in the odd-numbered conditions) and the other, the version with speech recognition (Version B, used in the even-numbered conditions). This way, within each difficulty level, the participant faced two different versions of the application. As a result, we were capable to obtain double the measurements from each difficulty level, by simultaneously avoiding boredom, which might have appeared if the participant played twice in each level, the exactly same version of the test.

#### Questionnaires

Stress self-assessment was conducted at the end of each condition (post-condition questionnaire), using two different question types. The first was a Likert-scaled (1–5) question directly asking subjects whether they were feeling stressed during the condition, following the rationale of the free-scale question used in [41]. The second was a subset of the Stress-Appraisal-Measure (SAM) questionnaire [43], consisting of the four questions related to stress (questions 2, 16, 24 and 26). Based on the two question types, two different variables were formed, to be thereafter used as ground truth (subjects' self-reported stress levels) in our analysis: 1) The answers that the subjects had given to the Likert-scaled question directly assessing stress (Stress_1–5) and 2) The average value of the subjects' answers to the four SAM questions (Stress_SAM).

#### Acquired Dataset

The acquired dataset consisted of 126 (21×6) trials that were recorded during subjects' playing the different versions of the stroop colour word test, 21 recorded during subjects' watching the relaxing video (condition 0), and also 21 rest sessions. Apart from the 21 rest sessions, in total, 147 trials were recorded. From all these relaxation and stroop-playing conditions, various features were extracted from each monitoring modality (GSR, ECG, video, accelerometer) and analyzed.

### Behavioural Features Extraction Procedure

In the current section, the extraction procedure of a plethora of activity-related features is described. Initially, the features that are solely based on visual information are presented, while the accelerometer-based behavioural features follow right after.

### Video-based Feature Extraction

**Figure 3:**
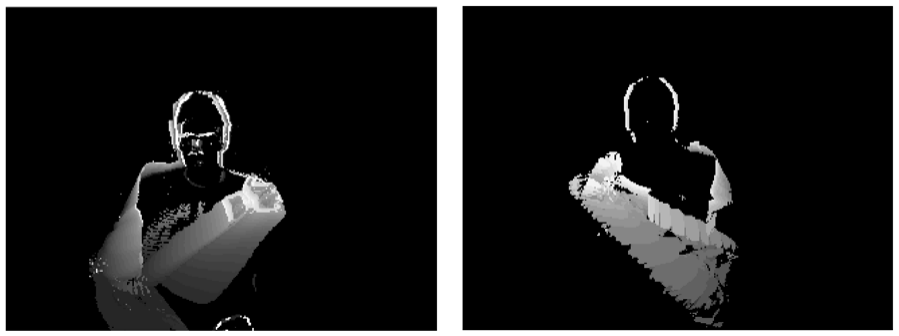
Samples of Motion History Images (MHIs) regarding activities “right hand to left shoulder” and “left hand to right shoulder”.

The vision based features examined in the current work are mainly based on spatiotemporal information of the subject's movements:

The proposed method for stress-related analysis of the user's movement is mainly based on Motion History Images (MHIs) [44], vision-based spatiotemporal descriptors that can be extracted from video sequences depicting the monitored subject (Figure 3). A MHI is a spatio-temporal template, where the intensity value (*MHI_T_*) at each point is a function of the motion properties at the corresponding spatial location in an image sequence, according to the following equation:
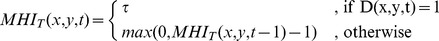
where τ is the number of frames contributing to the MHI generation and D(x,y,t) equals to 1 if there is a difference in the intensity of a pixel between two successive frame. The older a change is, the darker its depiction on the MHI will be, while changes older than τ frames faint completely out.

Before presenting each extracted feature, a short description of the MHI properties should be given. In this respect, it can be noticed in the equation above that the value of τ provides a notion about the history information that is taken into account. As such, large values of τ form an MHI that extends deep in the past, while small values refer only to the very recent past. Moreover, it is obvious that the bigger the differences between two successive frames, the larger the non-black area (A_non-black_≡A_nb_) on the MHI. Similarly, identical successive frames would produce a completely black MHI. This would be valid for any arbitrary number τ of utilized frames. Based on these observations, significant motion-related information can be extracted from an MHI, by properly adjusting parameter τ.

Given the specifications of our system (Intel i5–2500k processor, 4 GB RAM), an experimentally detected average frame rate value was 10 frames per second (fps) for online processing. Moreover, it was noticed that that human's small movements typically do not last longer than 1 sec. In this respect, the extracted (Long term -) MHIs are generated within the time period of 1 sec (∼10 frames) and updated accordingly. However, since a minimum duration for such movements cannot be trivially defined, and given the fact that fast and sudden movements may form a strong stress indicator, a second (Short term -) MHI is also produced in parallel, by processing only two successive frames (τ = 2).

In order to preserve a common reference for all subjects, the head's position is constantly tracked and updated by the system. The robust detection of the head's position is a vital prerequisite for the extraction of a series of stress related features, as it will be shown in the following. As such, in the current work the head detection algorithm was implemented as the combination of a face detection algorithm [45] and a tracking mean-shift based algorithm [46]. This enhancement is used, as the Haar-based detector utilised in Viola & Jones algorithm fails to detect a face, when the latter is significantly diverged from the frontal (camera) view.

Specifically, within our method, the face –and thus the head– centre is initially detected at each frame via the Viola & Jones's algorithm. If this algorithm fails, the last successfully detected face-rectangle with pixels 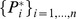 and centre P_0_ is passed to the mean-shift algorithm, and handled as follows:

First, a function b is defined: R_2_→1,…,m, which associates the pixel at location 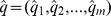 to the index b(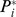) of the histogram bin corresponding to the colour of that pixel. The probability of a colour *u* in the target model is derived by employing a convex and monotonically decreasing kernel profile k, which assigns a smaller weight to the locations that are further away from the centre of the target. Therefore, it can be written as 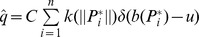, whereby C is computed by imposing the condition 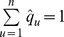, i.e. the summation of delta functions for u = 1,…,m is equal to one. Further, when the target model is passed on to the next frame, the probability p_u_ of colour u in the target candidate with a centre P_0_ and a radius h is calculated as:
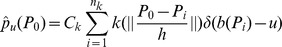
The most probable location P_0_ of the target pixel area in the current frame is obtained by minimizing the distance d(P′) at a given location y,
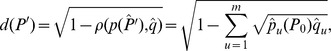
for 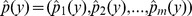 and 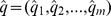, or by simply maximizing ρ(P′), the Bhattacharyya coefficient,
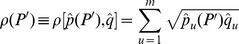



Based on the outcome of the aforedescribed MHI extraction and head detection algorithms, a series of behavioural features (**V1**, **V2**, **V3**,…) were defined and extracted from the video sequences that were recorded during the experiment. For better notation, these behavioural features can be regarded to form the video-based feature vector of our study: **F_v_** = {**V1**, **V2**,…}. The features that were extracted from the video modality belong to the following categories:

#### Global Activity Level

First of all, the Average and Standard Deviation (SD) of global upper-body Activity Level within a time period were examined as potentially useful behavioural features towards automatic stress detection. The specific features followed the rationale of [22], where the overall energy spent by a subject, approximated by the total amount of displacement in her/his hands and head, was examined as an expressive feature useful for automatic affect recognition. Also, in [29], it was found that dynamics/energy/power of movements can show significant differences among different emotional states.

Hence, the amount of non-black areas in the MHIs of a given time period was expected to provide a powerful clue of stress indication, given that stressed persons would probably move nervously (and arbitrarily), more frequently than calm ones. In this respect, a MHI regarding the last ten captured frames was constantly updated, and given this, the proportion k of the non-black area A_nb_ over the whole area A of an MHI was calculated as:
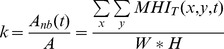
where W and H stand for the width and height of the MHI, respectively.

Based on the mean (Avg) and standard deviation (SD) of k within a condition's MHIs, various stress-related features were extracted: **V1**: Avg(k), **V2**: Avg(k), for k>0, **V3**: SD(k), for k>0. Moreover, each time the parameter k of a certain MHI exceeded the experimentally set threshold l (k>l), a signal “Increased Movement Detected” was triggered. This way, a new subset of the original MHIs was also preserved, that consisted only of executed movements above an energy threshold, so as to discard small-scale movements of the hands or head. Thus, the following features were extracted similarly to the above: **V4**: Avg(k) for MHIs with k>l and **V5**: SD(k) for MHIs with k>l. Moreover, similar features like **V4** and **V5** were extracted, by considering only the MHIs where the activity level exceeded a threshold smaller than the one used for “Increased Movement” detection (k>l_s_, 0<l_s_≪l). With this threshold, only micro-movements of extremely small scale (e.g. movement of the mouse of a PC) were discarded: **V6**: Avg(k) for k>l_s_, and **V7**: SD(k) for k>l_s_.

Finally, the frequency of the detected “Increased Activity Level” movements within the given time period was extracted as a further potentially stress-related feature with **V8**: The proportion of seconds with “Increased Movement” detected to the total number of seconds of the condition. The nominator used in feature **V8** calculation was taken as the number of non-overlapping seconds within the time period considered, for which at least an MHI with k>l existed. Feature **V8** aimed to provide a further indicator of the subject's activity level, by taking into account activities that produced MHIs denoting increased activation, and finally expressing their frequency within the examined time period.

#### Sharp Activities Energy

A *sharp* activity is defined within our proposed system as activity occurring between two consecutive recorded frames. Taking into account the definition of the MHIs, by restricting the analysis window (threshold τ) to the value of 2, MHIs can be extracted on the basis of only two consecutive frames (Short-term MHIs). Thus, the main difference of the features explained in the following of this section, compared to features **V1**, **V3**–**V5**,is the selection of parameter τ, which defines the inspection time for the generation of a single MHI; contrary to above, where τ had been set to 10 frames, a value of τ = 2 is hereby used for the current features.

Practically, this means that only the movement captured within two successive frames is taken into account. In this respect, rather rapid movements are expected to be detected, and from the MHIs, features expressing several qualitative characteristics (e.g. energy) of these rapid movements can be extracted. The larger the area of A_nb_, the faster can be considered the performed movement. Thus, the correlation between rapid, nervous movements and stress is attempted to be studied through the average of the proportion k_s_ of the non-black area (A_nb_) over the whole area A of the Short-term MHIs: **V9**: Avg(k_s_). Also, by considering only the short-term MHIs (τ = 2) corresponding to time periods where movement was detected (k>0) in the long-term (τ = 10) MHIs (as defined in feature **V2**), features **V10**: Avg(k_s_), for k>0 and **V11**: SD(k_s_), for k>0 were extracted. Finally, taking into account only the Short-term MHIs (τ = 2) that correspond to the Long-term ones (τ = 10) for which “Increased Movement” was detected (k>l), features **V12**: Avg(k_s_), for k>l and **V13**: SD(k_s_), for k>l, were extracted.

#### Activity Symmetry

**Figure 4:**
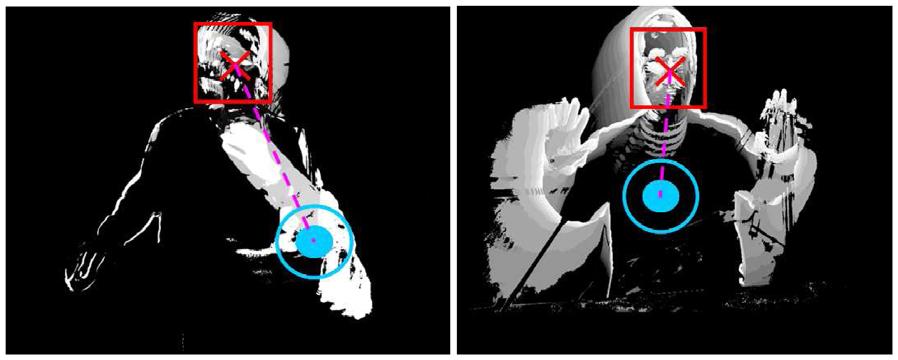
Non symmetric (left) and symmetric (right) action samples.

The relevance of gestural symmetry as behavioural and affective features has been recently studied [22]. Although in [22], no significant differentiation was found between several symmetry-related features and the quarters of the valence-arousal space, in [47], it was shown that that arm-position asymmetry was a relevant behavioral feature to identify a “relaxed” attitude and relative high social status of a person within a group. Following this line, activity symmetry-related features were extracted in our work from the MHIs of the recorded video sequences. In particular, the symmetry of the human gesture was defined as the divergence of the vector **s_v_**, drawn between the user's head and the MHI's centre of gravity from the upright position (Figure 4). Specifically, given that the head's location is detected as described above, its movements can be “subtracted” from the MHI. Then by estimating the centre of gravity (CoG) on the remaining MHI,
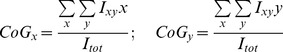
where I_xy_ stands for the image intensity at position (x, y) and 
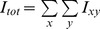
. Following this, one can draw the symmetry vector of the gesture (Figure 4):
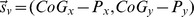



From the symmetry vectors of the MHIs taken within a time period of interest (i.e., a given condition of the experimental session), several features were extracted, by taking into account either all MHIs of the time period, or only those MHIs where *k* was larger than *l*, or *l_s_*, as shown in Table 1.

**Table 1:** Features extracted on the basis of the symmetry vector.

Formula	Feature Name		
	*All MHIs*	*MHIs with k>l*	*MHIs with k>l_s_*
Avg{S_v_(x)}	**V14**	**V22**	**V30**
Avg{S_v_(y)}	**V15**	**V23**	**V31**
Avg{S_v_(x)/|S_v_(y)|}	**V16**	**V24**	**V32**
Avg{Sqrt(S_v_^2^(x)+S_v_^2^(y))}	**V17**	**V25**	**V33**
SD{S_v_(x)}	**V18**	**V26**	**V34**
SD{S_v_(y)}	**V19**	**V27**	**V35**
SD{S_v_(x)/|S_v_(y)|}	**V20**	**V28**	**V36**
SD{Sqrt( S_v_^2^(x)+S_v_^2^(y))}	**V21**	**V29**	**V37**

From Table 1, it is clear that the extracted symmetry-related features mainly encode the average and standard deviation of the Euclidian, horizontal, and vertical size of S_v_, as well as its divergence from the upright position.

#### Position and movement of subject's head and MHI barycenters

According to the relevant literature [22], [29], [48], head position (sometimes indicative of pose) and movement can be considered as important features for distinguishing between various emotional expressions. Along this line, a set of features were extracted, expressing the position and movement of the subject's head during each condition. First of all, the Average (**V38**) and Standard Deviation (**V39**) of the head's distance from the image centre (IC) were calculated from: 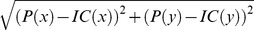. Additionally, the features shown in Table 2 were extracted by taking into account the initial position (IP) of the head within the same condition, the initial position of the head within the specific subject's condition 0 (IP_0), or the average position of the head within the specific subject's condition 0 (AP_0).

**Table 2:** Features extracted for expressing position and movement of subject's head.

Formula	Feature name		
	*P_0_ = IP*	*P_0_ = IP_0*	*P_0_ = AP_0*
Avg{P(x)−P_0_(x)}	**V40**	**V46**	**V52**
Avg{P(y)−P_0_ (y)}	**V41**	**V47**	**V53**
Avg{Sqrt( (P(x)−P_0_(x))^2^+(P(y)−P_0_ (y) )^2^) )}	**V42**	**V48**	**V54**
SD{ P(x)−P_0_ (x) }	**V43**	**V49**	**V55**
SD{ P(y)−P_0_ (y) }	**V44**	**V50**	**V56**
SD{ Sqrt( (P(x)−P_0_(x))^2^+(P(y)−P_0_ (y) )^2^) )}	**V45**	**V51**	**V57**

Also, the Average and SD of the head's velocity (**V58**, **V61** respectively), acceleration (**V59**, **V62**) and jerk (**V60**, **V63**) were calculated. Within our explorative study, these parameters were also examined in respect of the MHI barycenters (Centre of Gravity - CoG), since in [22], gestural smoothness/jerkiness were examined as behavioural parameters related to emotions. As a result, further features expressing qualitative aspects of the MHI barycenters' movement were extracted, as shown in Table 3.

**Table 3:** Features extracted for expressing position and movement of MHI barycenters.

Formula	Feature name	
	*MHIs with k>l*	*MHIs with k>l_s_*
Avg{Velocity(CoG)}	**V64**	**V70**
Avg{Acceleration(CoG)}	**V65**	**V71**
Avg{Jerk(CoG)}	**V66**	**V72**
SD{Velocity(CoG)}	**V67**	**V73**
SD{Acceleration(CoG)}	**V68**	**V74**
SD{Jerk(CoG)}	**V69**	**V75**

#### Frequency of specific gesture occurrence

From the MHIs, specific gestures made by the subject can also be detected. For this purpose, each recorded MHI was initially transformed according to the Radial Integration Transform (RIT) and the Circular Integration Transform (CIT), which were used due to their aptitude to represent meaningful shape characteristics.

**Figure 5:**
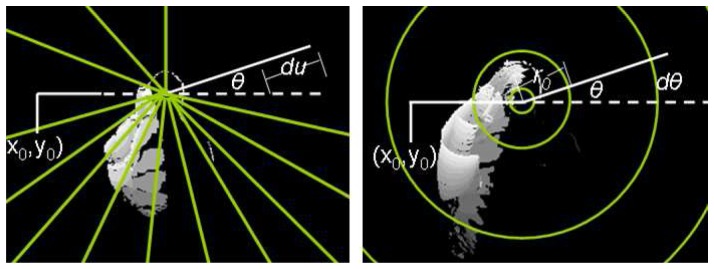
Illustration of the Activity detection algorithm using MHIs. Application of the RIT (left) and CIT (right) algorithms.

The RIT transform of a function f(..,..) is defined as the integral of f(..,..) along a line starting from a centre (x_0_, y_0_), which forms angle θ with the horizontal axis, (Figure 5, Left picture). In the proposed feature extraction method, the discrete form of the RIT transform was applied, which computes the transform in steps of Δθ and is given by equation:
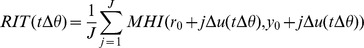
for t = 1,…,T with T = 360°/Δθ, where Δθ and Δu are the constant step sizes of angle θ and distance u, respectively. J is the number of pixels that coincide with the line of orientation R and are positioned between the centre of the head and the end of the MHI in that direction (Figure 5, Right).

In a similar manner, the CIT is defined as the integral of a function along a circle curve with centre (x_0_, y_0_) and radius ρ (Figure 5, Right). Similarly to the RIT transform, the discrete form of the CIT transform was used, as given by the following equation:
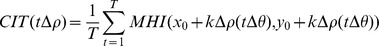
for k = 1,…,K with T = 360°/Δθ, where Δρ, and Δθ are the constant step sizes of the radius ρ and angle θ variables. Finally, kΔρ is the radius of the smallest circle that encloses the gray scaled MHI.

**Figure 6:**
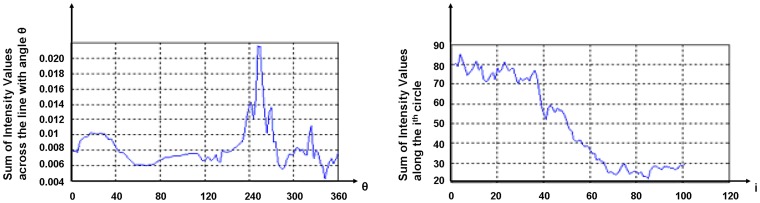
1D RIT Transform of the left image of Figure 5 (left) and 1D CIT Transform of the right image of Figure 5 (right).

Thus, each MHI can be represented by two 1 Dimensional vectors, which are simpler to process (Figure 6). It should be noted that the origin point (x_0_, y_0_) for the aforementioned transforms was taken in our case as the centre of the head, which was detected by the aforedescribed head detection algorithm.

Via the RIT- and CIT- based MHI transformations, specific gestures of the subject were detected, by applying a threshold-based template matching algorithm to pre-defined templates of gestures of interest. For this purpose, we created a gallery of pre-defined template MHIs, where one MHI existed for each gesture of interest. As gestures of interest we selected the “Right hand on head” and “Left hand on head” activities. These specific gestures were used due to the fact that activities like nail biting, scratching of head, smoothing of (already smooth or even long gone) hair etc. are known to occur as behavioural symptoms of stress [1].

In order to detect the gestures of interest, two matching scores between the probe and the gallery templates were simultaneously produced so as to increase both the robustness and the performance of the algorithm. These matching scores were a) the L1-Norm distance and b) the correlation factor between each of the RIT and CIT transformed vectors, as shown below:
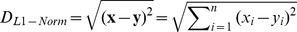


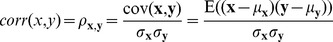



An event was detected, when only the returned scores from both classifiers exceeded experimentally selected thresholds, so as to diminish false positives. The final decision about which activity was occurring, was taken according to the most matches with the prototype MHIs within a predefined time-period (*majority voting rule*); in our case, this period was one second, and the analysis was performed in non-overlapping intervals.

Once the specific gestures had been detected within each non-overlapping one-second interval of the whole time period considered (i.e. the time period of a recorded condition), the following features were extracted, expressing the frequency of each specific gesture occurrence: **v76_RHH**: The ratio of the number of seconds with right hand raised towards head detected to the total duration (in seconds) of the condition, and **v77_LHH**: The ratio of the number of seconds with Left Hand raised towards head detected to the total condition's duration.

### Accelerometer-based Feature Extraction

Two tri-axial accelerometers were used in our framework, one at each knee of the participant. The aim was to monitor the occurrence of “foot trembling”, a behaviour known to often accompany stress. Each accelerometer provided a triplet of values denoting the acceleration in the three axes. Accelerometer data was collected with 60 Hz sampling rate and was processed in one second long, non-overlapping intervals.

Initially, within each interval, the total Power Spectral Density (PSD) of the accelerometer output was calculated, as the average PSD of the three axes. Following a rationale similar to [49], foot trembling was detected only when a) the proportion of signal power that existed in the experimentally set range [f_low_, f_high_] Hz to the total signal power and also b) the total signal power were both above experimentally selected thresholds. Each second of the recording where foot trembling was detected was annotated as such. Then, in order to diminish false positives, intervals were treated as pairs of consecutive ones; only when foot trembling was detected in both two consecutive intervals, these intervals were finally marked as having foot trembling occurrence. This processing was done for each of the two accelerometers separately, and eventually, the outcomes of the two accelerometers were fused by using an “*OR*” rule for each interval.

Following this processing, one feature was finally calculated from the accelerometer modality, which regarded the frequency of foot trembling occurrences within the time period of each condition: **A1**: The number of seconds with foot trembling detected to the total Nr of seconds.

### Physiological features extraction

A further set of features were also extracted from the biosignals (GSR, ECG) that were monitored throughout the experiment. This set consisted of features commonly used in the literature towards automatic stress, or in more general, affect detection. Given the acknowledged effectiveness of these biosignal features in our context, they were used to provide the basis for assessing whether the examined behavioural features are capable to enhance the accuracy of a typical automatic stress detection system. In the following, the features extracted from the GSR and ECG signals recorded during each trial (trials 0–6) of the experiment are described. GSR and ECG data were collected with 16 Hz and 256 Hz sampling rates, respectively. From the ECG data, Inter - Beat - Intervals (IBIs) were calculated directly from the monitoring device software. In order to treat between-subject variability in physiological measurements, all extracted biosignal features were normalized by division to their baseline values, recorded during each subject's rest session.

*From both the GSR and IBI time series*, the following typical features were extracted for each trial: Average (**Avg**) and Standard Deviation (**SD**) [18], Minimum (**Min**) and Maximum (**Max**). Moreover, following [15], the mean of the absolute values of the first differences of the raw and normalized signals were calculated:
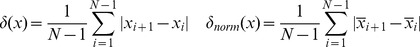



Also, the mean of the convoluted with a Hanning window GSR and IBI signal first differences were given by:
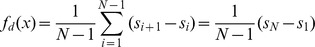



In the above three equations, *x* is the IBI or GSR signal, *s_i_* is the *i^th^* sample of the resulting time series of the raw signal, sub-sampled at 16 Hz and convoluted with a 3-second Hanning window. As in [15], the normalized signal 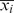 used in *δ_norm_(x)* calculation, was given by (x_i_−x_mean_)/x_sd_, where *x_i_* is a signal value recorded during a trial, *x_mean_* and *x_sd_* are the signal's average and standard deviation during the trial, respectively. Moreover, the Skewness and Kurtosis [50] of the GSR and IBI signals were calculated by:
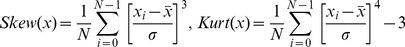
where *x* is the IBI or GSR signal, *x_i_* is the *i^th^* sample of the raw signal, 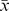 and *σ* are the signal's average and standard deviation, respectively.

Furthermore, the following features were extracted *only from the GSR signals* recorded during the trials: Average, RMS (Root Mean Square), and proportion of negative samples of the 1^st^ Derivative **(Avg1, RMS1, prop1)** and the smoothed (convoluted with Bartlett window) 1^st^ Derivative **(Avg1s, RMS1s, prop1s)** following [51]. Skin Conductance Responses (SCRs) were detected similarly to [51], and their Average Amplitude and Duration (**SCR_Amp**, **SCR_Dur**) were calculated for each trial. Also, the Rate of SCR occurrences (number of SCRs divided to the intermediate duration, **SCR_Rate**), as well as Quantile thresholds at 25%, 50%, 75%, 85%, and 95% for Amplitude (**SCR_AmpQ25**, …, **SCR_AmpQ95**) and Duration (**SCR_DurQ25**, …, **SCR_DurQ25**) of SCRs were calculated similarly to [42]. Furthermore, the average area under the rising half of each GSR response (**SCR_arUnder**) was calculated.

*From the ECG modality and the IBI time series* of each trial, the following features, typically used in the literature towards biosignals-based stress and affect detection were extracted: **RMSSD**, **pNN50**, average LF/HF power ratio (**LF/HF**). Also, the standard deviations **SD1** and **SD2** were calculated from the IBI Poincare' plot geometry similarly to [52]. Finally, following [15], the mean of the absolute values of the second differences of the raw normalized signals were calculated by:
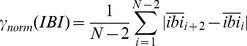



## Data Analysis and Results

### Stressor Effectiveness

**Figure 7:**
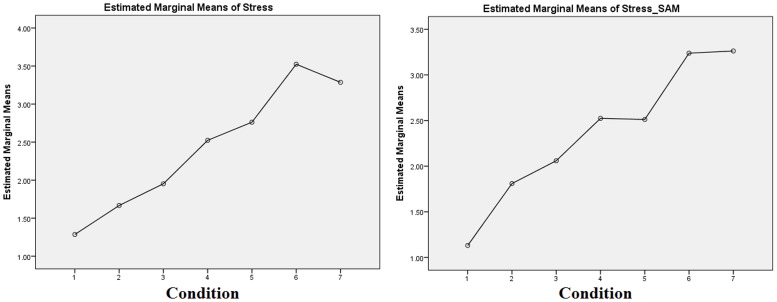
Average per condition self-reported stress values showing that stress increased at increasing task difficulties.

Figure 7 shows the mean values of all participants' answers to the stress self-assessment questions (Stress_1–5 and Stress_SAM variables, as defined in section *“Questionnaires”*), in respect of the different conditions of our experiment (specific values are given in Table 4 below). A linear regression showed an increase of self-reported stress (Stress_1–5) with the condition (β = 0.376, S.E. = 0.036, p<.001, Adjusted R Square = 0.429) and an increase of SAM Questionnaire index (Stress_SAM) with the condition (β = 0.347, S.E. = 0.034, p<.001, Adjusted R Square = 0.419). As can be seen from Figure 7, the stressor employed in our experiment was eventually found to be effective (especially in condition 5), leading to average stress self-reporting values close to relevant ones that have been reported in the literature [41].

**Table 4:** Average per-condition values of a) responses to stress self assessment questions, b) physiological features and c) behavioural features.

	Mean (St. Error)						
Condition	0	1	2	3	4	5	6
Stress_1–5	1.286 (0.101)	1.667 (0.174)	1.952 (0.146)	2.524 (0.225)	2.762 (0.206)	3.524 (0.203)	3.286 (0.23)
Stress_SAM	1.131 (0.051)	1.81 (0.175)	2.06 (0.151)	2.524 (0.189)	2.512 (0.18)	3.238 (0.202)	3.262 (0.237)
Avg(GSR)	1.253 (0.091)	1.854 (0.194)	2.528 (0.468)	2.679 (0.273)	3.356 (0.396)	3.65 (0.42)	3.922 (0.511)
SD(GSR)	1.288 (0.274)	1.302 (0.385)	1.795 (0.477)	2.839 (0.747)	3.064 (0.701)	3.276 (0.73)	3.443 (0.786)
SCR_DurQ25	0.266 (0.142)	0.352 (0.253)	0.368 (0.151)	0.548 (0.338)	0.901 (0.33)	1.779 (0.934)	1.512 (0.857)
Avg(IBI)	0.994 (0.007)	0.954 (0.015)	0.96 (0.013)	0.914 (0.017)	0.915 (0.02)	0.919 (0.018)	0.943 (0.018)
SD(IBI)	1.334 (0.223)	0.596 (0.046)	0.71 (0.121)	0.895 (0.136)	0.772 (0.068)	0.991 (0.145)	0.9 (0.073)
RMSSD	1.681 (0.321)	0.501 (0.053)	0.81 (0.287)	1.069 (0.335)	0.54 (0.09)	0.995 (0.204)	0.963 (0.198)
pNN50	1.304 (0.225)	0.686 (0.123)	0.41 (0.086)	0.783 (0.133)	0.884 (0.244)	1.716 (0.878)	0.888 (0.142)
LF/HF	1.462 (0.259)	2.005 (0.394)	2.264 (0.469)	1.572 (0.37)	2.404 (0.433)	1.836 (0.406)	2.539 (0.634)
δ(IBI)	1.465 (0.287)	0.773 (0.052)	0.769 (0.102)	0.854 (0.084)	0.714 (0.051)	1.163 (0.247)	0.871 (0.071)
f_d_(IBI)	0.792 (0.074)	0.007 (0.117)	0.124 (0.072)	0.159 (0.043)	0.143 (0.049)	0.149 (0.034)	0.146 (0.058)
Min(IBI)	0.817 (0.066)	0.972 (0.034)	0.929 (0.044)	0.816 (0.043)	0.796 (0.054)	0.728 (0.063)	0.72 (0.058)
V1	0.001 (0)	0.001 (0)	0.002 (0)	0.002 (0)	0.002 (0)	0.002 (0)	0.003 (0.001)
V5	0.006 (0.001)	0.005 (0.002)	0.006 (0.001)	0.024 (0.002)	0.024 (0.001)	0.026 (0.001)	0.025 (0.001)
V7	0.007 (0.001)	0.007 (0.002)	0.007 (0.001)	0.016 (0.001)	0.015 (0.001)	0.016 (0.001)	0.015 (0.001)
V8	0.038 (0.008)	0.026 (0.006)	0.059 (0.011)	0.045 (0.005)	0.068 (0.009)	0.07 (0.01)	0.109 (0.023)
V16	−0.054 (0.193)	0.576 (0.081)	0.525 (0.228)	0.685 (0.08)	0.301 (0.133)	0.703 (0.069)	0.361 (0.168)
V31	0.142 (0.023)	0.1 (0.018)	0.096 (0.018)	0.081 (0.012)	0.054 (0.009)	0.071 (0.008)	0.06 (0.013)
V46	0.074 (0.037)	0.056 (0.037)	0.06 (0.037)	0.045 (0.038)	0.055 (0.038)	0.045 (0.037)	0.053 (0.038)
V52	0 (0)	−0.019 (0.005)	−0.015 (0.005)	−0.029 (0.009)	−0.019 (0.005)	−0.03 (0.005)	−0.021 (0.005)
V54	0 (0)	0.048 (0.008)	0.051 (0.009)	0.06 (0.014)	0.055 (0.009)	0.06 (0.008)	0.068 (0.01)
A1	0.004 (0.003)	0.011 (0.011)	0.037 (0.033)	0.013 (0.008)	0.052 (0.034)	0.107 (0.048)	0.068 (0.038)

### Correlations between behavioural features and stress

The hierarchical structure of the experiment data makes traditional forms of analysis less resilient to the different levels considered. Subjects are measured repeatedly, at many time points. Traditional repeated-measures designs require the same number of observations for each subject and no missing data, being thus suitable for our case. However multilevel models are appropriate to analyze such data, above all, because the existent dependencies due to repeated measurements are included in the parameter estimates. Moreover, further dependencies existing in the data can be taken into account.

Since in our case, the entries were nested within the conditions and within participants, physiological and behavioural indexes were estimated on stress level extracted through the free scale (Stress_1–5) questionnaire, with hierarchical linear analysis, an alternative to multiple regression, suitable for our nested data. We referred to two levels in the model: condition-level and subject-level.

Selection of the models was done on the basis of three criteria:

1. significance levels of involved variables;
2. Quasi Likelihood under Independence Model Criterion (QIC) in the smaller-is-better form;
3. Corrected Quasi Likelihood under Independence Model Criterion (QICC) in the smaller-is-better form.

The usual goodness of fit statistics, like R-square, could not be computed. Instead, the above information criteria, based on a generalization of the likelihood were computed.

In particular, using self-reported stress as our dependent variable, analyses consisted of:

1. A mixed linear hierarchical regression for each variable in the dataset, namely 78 behavioural features, 29 GSR and 15 ECG features.
2. Selection on the basis of Step 1 results, using significance levels, QIC and QICC.
3. A mixed linear hierarchical regression for ECG features: δ(IBI), f_d_(IBI), Min(IBI)
4. A mixed linear hierarchical regression for GSR features: SD(GSR), SCR_DurQ25
5. A mixed linear hierarchical regression for behavioural features: V5, V16, V31, V46
6. A mixed linear hierarchical regression for all features at points 3–5
7. Selection on the basis of Step 6 results, using significance levels, QIC and QICC.
8. A mixed linear hierarchical regression for all consistent variables: V5, V16, V31, V46, SCR_DurQ25, f_d_(IBI).

Table 5 shows the mixed linear hierarchical regressions conducted per each feature (independent variable) using self-reported stress as the dependent variable; in particular, results for features selected from Step 2 of the aforementioned analysis are presented. From Table 5, it is evident that significant effect of stress was found for several physiological, as well as behavioural features. As concerns behavioural parameters, a positive relation was found between stress and features expressing aspects of the *Global Activity Energy* (**V1**, **V2**, **V4**, **V5**, **V7**, **V8**) and the *Sharp Activities Energy* (**V9**, **V10** and **V11**). Significant stress effect was also found for features focusing on *Activity Symmetry* (**V14**, **V16**, **V31**, **V33**), as well as ones related to the *Position and movement of the head* (**V41**, **V46**, **V52**, **V54**, **V58**, **V59**, **V60**) and the *MHI barycenters* (**V70**). Interestingly, increased self-reported stress was also found to be accompanied with increase in the frequency of occurrence of *Right Hand on Head movements* (**V76**) and *foot trembling* (**A1**).

**Table 5:** Mixed hierarchical regression per index (dependent variable: Stress_1–5).

Parameter Estimates									
Physiological Parameter	B	Std. Error	Hypothesis Test		Behavioural Parameter	B	Std. Error	Hypothesis Test	
			Wald Chi-Square	Sig.				Wald Chi-Square	Sig.
					V1	261.958	61.5407	18.119	.000
***GSR***					V2	119.459	45.4592	6.906	.009
Avg	.423	.1227	11.887	.001	V4	38.370	14.6119	6.896	.009
SD	.209	.0443	22.195	.000	V5	58.610	8.6949	45.437	.000
SCR_Amp	.004	.0016	6.746	.009	V7	80.222	24.9997	10.297	.001
SCR_arUnder	.0004	.0001	17.612	.000	V8	7.091	2.0560	11.897	.001
δ(GSR)	4.541	.9431	23.187	.000	V9	1147.67	312.81	13.461	.000
δ_norm_(GSR)	−.178	.0733	5.874	.015	V10	608.184	219.02	7.711	.005
Min	.444	.0895	24.612	.000	V11	330.460	152.77	4.679	.031
Max	.433	.1106	15.308	.000	V14	.005	.0020	6.969	.008
Skew	−.816	.3603	5.135	.023	V16	.336	.1075	9.739	.002
SCR_AmpQ95	2.160	.8487	6.477	.011	V31	−3.755	1.0641	12.453	.000
SCR_DurQ25	.171	.0410	17.410	.000	V33	−4.824	1.1179	18.625	.000
RMS1s	.024	.0053	20.052	.000	V41	−1.601	.7294	4.819	.028
					V46	−1.574	.4363	13.021	.000
***ECG***					V52	−16.233	2.7698	34.347	.000
pNN50	−.053	.0247	4.537	.033	V54	9.644	1.7730	29.587	.000
δ(IBI)	−.213	.0768	7.713	.005	V58	49.051	8.3143	34.806	.000
δ_norm_(IBI)	−.681	.3138	4.715	.030	V59	3.643	.539	45.686	.000
γ_norm_(IBI)	−.504	.2440	4.259	.039	V60	.179	.0338	28.097	.000
f_d_(IBI)	−.605	.1953	9.585	.002	V70	−8.999	3.8656	5.419	.020
Min	−.674	.2762	5.959	.015	V76	16.625	5.2868	9.888	.002
					A1	2.177	.8195	7.055	.008

Table 6 shows the four mixed linear hierarchical multiple regression models, conducted per different combinations of multiple independent variables, using again self-reported stress as the dependent (Steps 3, 4, 5 and 8). Features **V5**, **V16**, **V31** and **V46** were found to have significant effect within the final, full model that consisted of both behavioural and physiological features (Table 6). It was thus found that features expressing the SD of the global activity level (**V5**), our proposed activity symmetry vector's divergence from the upright position (**V16**) and vertical length (**V31**), as well as the horizontal difference between the head's position during the trial and its position during relaxation (**V46**), had significant effect in modelling stress, even when used in conjunction to physiological features derived from the GSR and ECG modalities.

**Table 6:** Models of mixed hierarchical regression (dependent variable: Stress_1–5).

Parameter Estimates					
				Hypothesis Test	
	Parameter	B	Std. Error	Wald Chi-Square	Sig.
Model GSRs	(Intercept)	1.731	.2790	38.472	.000
	SD(GSR)	.235	.0475	24.381	.000
	SCR_DurQ25	.185	.0402	21.147	.000
	(Scale)	1.134			
Model ECGs	(Intercept)	3.913	.3471	127.137	.000
	δ(IBI)	−.360	.0944	14.507	.000
	f_d_(IBI)	−.522	.1938	7.249	.007
	Min(IBI)	−1.250	.3063	16.649	.000
	(Scale)	1.195			
Model Gestures	(Intercept)	1.698	.1914	78.699	.000
	V5	52.964	9.0746	34.066	.000
	V16	.284	.1037	7.475	.006
	V31	−1.899	.9076	4.379	.036
	V46	−1.892	.4072	21.582	.000
	(Scale)	.777			
Full Model	(Intercept)	1.815	.2301	62.204	.000
	V5	56.123	8.4553	44.058	.000
	V16	.288	.1125	6.549	.010
	V31	−2.382	1.0186	5.470	.019
	V46	−1.740	.4281	16.516	.000
	SCR_DurQ25	.059	.0348	2.855	.091
	f_d_(IBI)	−.660	.1622	16.545	.000
	(Scale)	.758			

**Figure 8:**
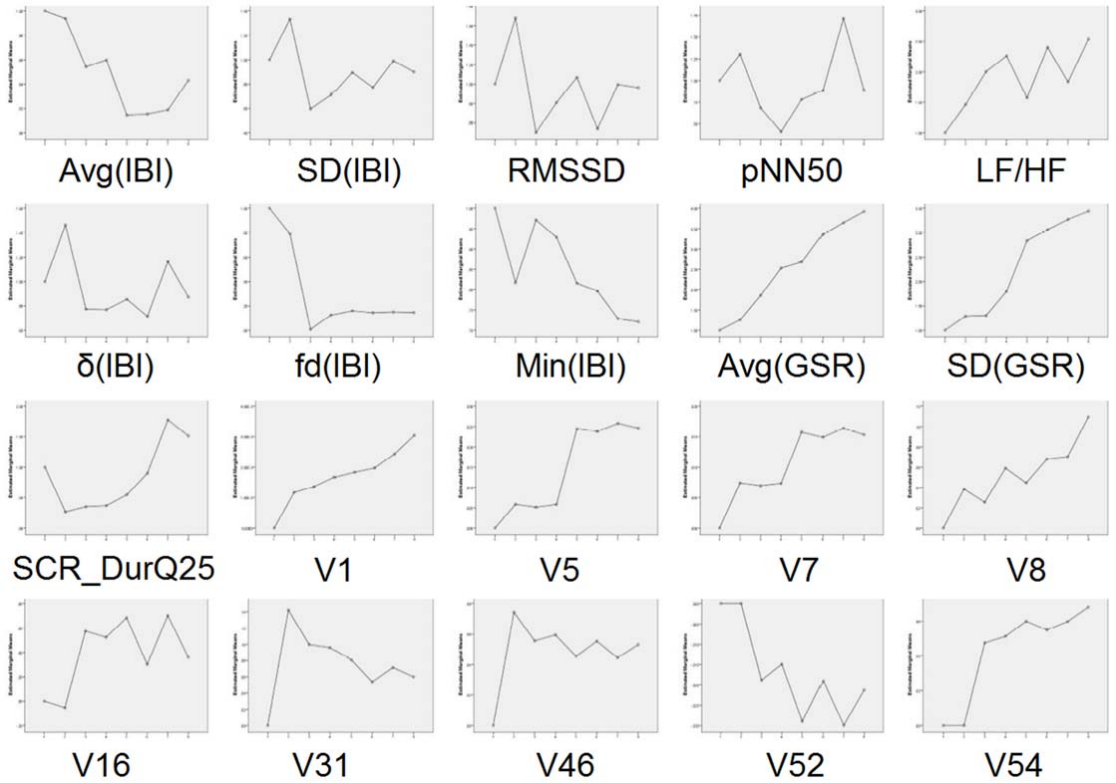
Variation of physiological and behavioural features among conditions.

Finally, Figure 8 (specific values are given in Table 4) depicts the variation of several of the examined variables among the different conditions. As shown from Table 6 and Figure 8, the results obtained can be regarded to confirm a relationship between physiological, behavioral and psychological data of our experiment.

### Efficiency of behavioural features towards automatic stress detection

From the analysis presented above, it is clear that some of the behavioural features showed significant relation to self-reported stress, similarly to physiological (GSR and IBI) features commonly used in the relevant literature (e.g. [41], [42]) towards automatic stress detection. Following these findings, it was examined whether the proposed behavioural features can be used in conjunction with (or even instead of) the typical physiological features, to enhance the effectiveness of automatic stress detection.

For this purpose, an LDA-based classifier [52] was used over the multi-subject data set of our experiment. In Fisher's LDA, the optimum projection for a given data set is realized through the transformation matrix ***W***, which is calculated so as to maximize the formula:
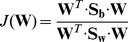
where **S_b_** is the “between class scatter matrix” and **S_w_** is the “within class scatter matrix” of the train data set. In two-class LDA, data from the initial feature space is projected on a single projection axis, which best discriminates training data among the available classes. Thus, once the optimum transformation vector ***W*** is calculated from the train data set, it can be used to calculate the projection of each class Centroid and each new (test) case to the transformation axis. Classification is then performed in the transformed space by assigning the new case to its less distant class found over the projection axis using:
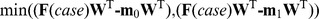
where **F**(case) is the feature vector of the test case, **m_0_** and **m_1_** are the centroids of the two classes under consideration, calculated using the training data, and **W** is the transformation matrix. Leave-one-out cross validation (LOOCV) was employed as in [52], and the final Correct Classification Ratio (CCR) of the classifier was calculated by CCR = N_c_/N, where *N_c_* is the number of cases correctly classified and *N* is the total number of cases constituting the full data set.

In order to assess whether behavioural features are useful towards automatic stress detection, various different feature sets consisting of physiological features and/or behavioural ones were used as the input of the classifier, in an effort to identify:

The effectiveness of well-known physiological features towards stress detection in the given dataset (used thereafter as a basis for comparison).
The effectiveness of the behavioural features towards stress detection, compared to the physiological features.
Whether there would be an increase (or decrease) in stress detection accuracy, using behavioural features auxiliary to physiological ones, compared to the initial stress detection accuracy achieved using only physiological features.

For each different feature set considered, an SBS (Sequential Backward Search) feature selection procedure was employed as in [52], to retain the subset of features that would yield in each case the best stress detection accuracy. By starting with a full, initial feature set, SBS initially calculated a criterion value, in our case, the classifier's performance. An iterative feature removal process was then employed, and within each iteration, the feature whose removal increased more the criterion value was definitely removed from the feature set. As a result, the features that produced the best CCR were finally selected from the initial feature set.

#### Data Annotation

The LDA-SBS classification schema was applied over two different two-class stress detection problems, which were formulated by following a different annotation procedure over the full dataset, which consisted of 147 cases in total (i.e., all recordings of all subjects regarding trials 0–6).

For the annotation of the first dataset (**Dataset1**), the subjects' answers to the Likert-scaled direct stress self-assessment question (Stress_1–5) were taken into account. In particular, trials for which the answer to this question was “1” or “2” were annotated as “Not Stressed” (*NS*), whereas trials for which this answer was “4” or “5” were annotated as “Stressed” (*S*) ones. Trials for which the specific answer was “3” were excluded. As a result, Dataset1 consisted of 108 trials in total, 82 labelled as *NS*, and 26 as *S*.

For the annotation of the second dataset (**Dataset2**), the average of the subjects' answers to the four SAM-questions (Stress_SAM) was taken into account. In particular, trials for which the average value of the answers to these questions was higher than 2.5 were annotated as “Stressed” (*S*), and the rest of trials were annotated as “Not Stressed” (*NS*). As a result, Dataset2 consisted of 147 trials in total, 93 labelled as *NS*, and 54 as *S*.

The purpose of using these two datasets was to evaluate the LDA-based classification schema on stress detection applied over:

A portion of the full dataset of this study, containing only the more extreme cases of not stressed trials and stressed ones (Dataset1)
The full dataset of this study (Dataset2)

For each dataset, three different feature sets were used as the initial feature set of the SBS procedure, consisting of:

All physiological features (feature set: *FS1*)
All behavioural features (feature set: *FS2*)
All physiological and all behavioural features (feature set: *FS3*)

From each feature set, SBS selected the features that provided the best CCR for each of the two different stress detection problems. In the following, the confusion matrices of the best stress detection results obtained from the various feature sets in respect of Dataset1 and Dataset2 are provided, along with the features that were finally selected from SBS and yielded the best results in each feature set case.

#### Classification Results

In respect of Dataset 1, as shown from a comparison between Tables 7 and 8, the behavioural features extracted proved equally effective to the physiological features in the stress detection problem concerning the more extreme cases of stress and no stress that existed in this dataset. Furthermore, when behavioural features were combined with the physiological ones, the best average CCR significantly increased (by 7.41%), achieving the maximum correct classification rate of 100% (Table 9).

**Table 7:** Confusion Matrix of the best feature set selected from FS1 (physiological features) in Dataset1.

	Classified as NS	Classified as S	total	class CCR
**NS**	**80**	2	82	97.56%
**S**	6	**20**	26	76.92%

Best Average CCR = **92.59%** (100/108).

Features Selected from *FS1*: SD(GSR), Avg1(GSR), RMS1(GSR), SCR_Rate, SCR_Amp, Min(GSR), Max(GSR), SCR_AmpQ75, SCR_AmpQ85, RMS1s(GSR), Avg(IBI), SD(IBI), δ(IBI), δ_norm_(IBI), f_d_(IBI), Max(IBI), Kurt(IBI), SD2(IBI).

**Table 8:** Confusion Matrix of the best feature set selected from FS2 (behavioural features) in Dataset1.

	Classified as NS	Classified as S	total	class CCR
**NS**	**78**	4	82	95.12%
**S**	4	**22**	26	84.62%

Best Average CCR = **92.59%** (100/108).

Features Selected from *FS2*: A1, V1, V2, V4, V10, V12, V16, V17, V22, V24, V29, V15, V27, V30, V34, V37, V36, V6, V41, V44, V47, V48, V51, V53, V57, V58, V61, V63, V70, V71.

**Table 9:** Confusion Matrix of the best feature set selected from FS3 (physiological and behavioural features) in Dataset1.

	Classified as NS	Classified as S	total	class CCR
**NS**	**82**	0	82	100%
**S**	0	**26**	26	100%

Best Average CCR = **100%** (108/108).

Features Selected from *FS3*: A1, V8, V5, V12, V13, V14, V17, V21, V25, V28, V15, V19, V23, V27, V30, V32, V33, V31, V35, V38, V39, V40, V42, V51, V52, V53, V54, V57, V58, V59, V62, V71, V72, V75, V65, V68, V69, Avg(GSR), Avg1(GSR), RMS1(GSR), SCR_Dur, SCR_arUnder, δ(GSR), prop1(GSR), Min(GSR), Max(GSR), SCR_AmpQ75, SCR_AmpQ85, SCR_AmpQ95, SCR_DurQ75, SCR_DurQ95, RMS1s(GSR), prop1s(GSR), Avg(IBI), RMSSD, pNN50, LF/HF, γ_norm_ (IBI), f_d_(IBI), Max(IBI), Kurt(IBI), Skew(IBI), SD1(IBI).

Regarding Dataset 2, by comparing Tables 10 and 11, it is clear that the proposed behavioural features appeared more effective than the physiological ones in the stress detection problem concerning all stress/no stress cases that existed in our dataset; a significant increase of 7.49% in the CCR was achieved from the behavioural features. The significance of this increase in performance was proved as in [53], by a two-tailed pair-wise t-test, applied over the classification results of FS1 and FS2 (t = 1.996, df = 146, p<.05). Moreover, when behavioural features were used together with the physiological ones as the initial feature set of SBS, the best average CCR again significantly (t = 3.964, df = 146, p<.001) increased (Table 12); by 13.61%, compared to the best average CCR achieved with physiological features.

**Table 10:** Confusion Matrix of the best feature set selected from FS1 in Dataset2.

	Classified as NS	Classified as S	total	class CCR
**NS**	76	17	93	81.17%
**S**	8	46	54	85.19%

Best Average CCR = 82.99% (122/147).

Features Selected from *FS1*: SCR_Dur, δ_norm_(GSR), prop1(GSR), Max(GSR), Skew(GSR), SCR_DurQ75, SCR_DurQ85, SCR_DurQ95, prop1s(GSR), RMSSD, LF/HF, f_d_(IBI), SD1(IBI).

**Table 11:** Confusion Matrix of the best feature set selected from FS2 in Dataset2.

	Classified as NS	Classified as S	total	class CCR
**NS**	**86**	7	93	92.47%
**S**	7	**47**	54	87.04%

Best Average CCR = **90.48%** (133/147).

Features Selected from *FS2*: A1, V8, V76_RHH, V77_LHH, V1, V2, V4, V5, V13, V14, V16, V17, V24, V29, V19, V23, V27, V33, V31, V34, V35, V37, V36, V38, V39, V43, V44, V46, V47, V48, V51, V52, V58, V62, V63, V70, V71, V72, V74, V70, V71, V67, V68, V69.

**Table 12:** Confusion Matrix of the best feature set selected from FS3 in Dataset2.

	Classified as NS	Classified as S	total	class CCR
**NS**	92	1	93	98.92%
**S**	4	50	54	92.59%

Best Average CCR = **96.60%** (142/147).

Features Selected from *FS3*: V8, V76_RHH, V77_LHH, V1, V2, V4, V3, V5, V14, V17, V18, V20, V22, V26, V15, V23, V32, V33, V31, V37, V36, V6, V38, V39, V41, V43, V45, V46, V47, V54, V57, V61, V70, V71, V66, V67, SCR_arUnder, δ_norm_(GSR), prop1(GSR), Max(GSR), SCR_DurQ95, prop1s(GSR), RMSSD, LF/HF, δ(IBI), f_d_(IBI), Max(IBI).

Furthermore, instead of using all features of our work, SBS and the LDA classifier was also applied only over the features for which significant regressions to self-reported stress levels were found, from the aforedescribed regression analysis (Table 5). The classification results obtained in respect of Dataset1 were: 88.89% for physiological (8 selected) features and 96.30% for physiological and behavioural features (13 selected features, 4 physiological and 9 behavioural). The respective results for Dataset2 were 73.47% with 6 features and 86.40% with 26 features (10 physiological and 16 behavioural). Behavioural features were thus again found effective in both datasets, even when the initial feature space of the SBS procedure was already limited through the regression analysis.

As said, all above classification analyses were based on LOOCV. Furthermore, in order to examine our approach over a completely independent validation sample than the training one, we randomly split the full dataset (consisting of data taken from 21 participants) into a training set, consisting of data taken from 9 participants and a validating one, consisting of data taken from the rest 12 participants. Using validation samples of subjects whose data was absolutely absent during training, simulates the hardest affect detection scenario, where the system tries to identify emotions of unknown persons, on the basis of knowledge it has taken from a limited training set. Overall stress detection accuracy was therefore expected to decrease in this case, however, the purpose of this analysis was to examine whether behavioural features would again lead to increase in automatic stress detection performance.

In respect of Dataset1, by applying SBS and training the LDA-based classifier with the training set, the physiological features provided stress detection accuracy of 72.88% (43/59; NS:39/46, S:4/13), whereas the joint use of physiological and behavioural features (FS3) achieved a CCR of 83.05% (49/59; NS:41/46, S:8/13). In Dataset2, the respective results were 66.67% (56/84; NS:44/56, S:12/28) and 78.57% (66/84; NS:47/56, S:19/28). As was expected, these results were in general inferior to the respective ones that had been obtained with LOOCV. However, the behavioural features were again found to significantly (t = 2.562, df = 58, p<.014 for Dataset1 and t = 3.349, df = 83, p<.002 for Dataset2) increase the performance that was achieved by using only physiological features, further underlining our proposed features' effectiveness and potential for future practical use.

### Using behavioural features to predict stress-related increase in the GSR

The analysis of the previous section focused on the automatic detection of stress, using self reports as the ground truth for the classification. It could however be argued that physiological responses (such as the increase in the average GSR level) could provide a more objective and reliable measure of stress than self reports. From this perspective, it would be interesting to also examine whether our proposed behavioural features could also be used so as to effectively predict stress-related alterations of physiological signals. Therefore, following the correlates that were found between behavioural features and physiological ones, a further analysis was conducted over the present dataset, towards assessing whether the increase in the average GSR level (a well-known, reliable index of stress [41]) could be predicted through the proposed features.

For this purpose, a further dataset was formed (Dataset3), by annotating the recorded conditions on the basis of the GSR average value (*Avg(GSR)*). In order to do so, a normalized value for the *Avg(GSR)* value of each recorded condition was calculated as:
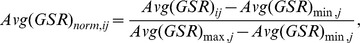
where *Avg(GSR)_norm,ij_* is the normalized GSR value of condition *i* of participant *j*, *Avg(GSR)_ij_* is the actual value of the *Avg(GSR)* feature for the same condition, *Avg(GSR)_min,j_* and *Avg(GSR)_max,j_* are the minimum and maximum values of the *Avg(GSR)* feature, found in all conditions of participant *j*. The result of the above equation was a value in the range [0–1], expressing the increase in GSR level that was observed within each subject's recorded conditions. Then, considering the average value of the GSR as the ground truth, annotation took place on the basis of *Avg(GSR)_norm_*, with the rule: If *Avg(GSR)_norm_*<0.5, the condition was labelled as “Not Stressed” (NS). Otherwise, the condition was labelled as “Stressed” (S). As a result, Dataset3 consisted of 147 cases in total, 57 annotated as NS and 90 as S.

Following this labelling process, Dataset3 had as ground truth, each participant's increase in GSR, instead of the answers given to stress self-reports. Thus, the purpose was in fact to examine whether the behavioural features can predict the increase in GSR, which can in turn be regarded as a reliable measure of stress. By applying LOOCV over the behavioural features (selected from FS2 feature set after SBS), stress (GSR increase) detection accuracy at the level of 93.88% (138/147; NS:54/57, S:84/90) was obtained. This result further underlines the correlation that exists between the behavioural features of our work and the level of GSR, a well-known physiological stress metric.

In the same context, a further analysis followed, using this time self-reported stress, so as to predict stress as measured by the increase in GSR. In this case, we considered two further features, the Stress_1–5 and the Stress_SAM, which regarded the respective self-reports that were obtained after the end of each recorded condition. When these two features were used in the LDA-based classifier, stress (increase in GSR) was predicted in Dataset3 with accuracy of 75.51% (111/147; NS:47/57, S:64/90). Thereafter, these two features formed together with the behavioural ones a further feature set (FS4), from which the best features (selected after SBS) provided stress detection accuracy in Dataset3, at the level of 94.56% (139/147; NS:55/57, S:84/90). From a perspective that takes as reference the subject's increase in stress level, as depicted from the increase in GSR, the latter two results indicate that our behavioural features, used together with self-reports, can lead to significant (t = 5.039, df = 146, p<.001) increase in the performance of a stress detection system that is solely based on self-reports.

## Discussion

In this work, a large set of seventy eight behavioural features were extracted from video and accelerometer data collected in the conducted experiment, and analysed with the aim to answer the two research questions of the present study.

Our first research question (RQ1) aimed to understand the relationship between automatically extracted behavioural features and self-reported stress levels of subjects. Analyses based on mixed linear hierarchical regression models appeared to confirm that relationships between the proposed behavioural features and self-reported stress exist. We defined statistical models of self-perceived stress, explained by the calibrated mixing of physiological and behavioural measures. This showed in an even more clear way, the subtle relationships among different changes in subjects' behaviour due to increased stress level. Interestingly, several behavioural features were found to have significant effect in modelling self-reported stress, even when used in conjunction to physiological features.

Our second research question (RQ2) aimed to investigate whether more robust stress detection is feasible by adding automatically detected behavioural information. Results showed that when the behavioural features were used together with common physiological measures (*FS3*), stress detection accuracy significantly increased, compared to the case when only the latter were utilized (*FS1*). It was even observed that in the full dataset of the present study (Dataset2), the total replacement of the physiological features from the proposed behavioural ones (i.e. using feature set *FS2* instead of *FS1*), led again to increase in performance. Moreover, behavioural features appeared to also enhance automatic stress detection within a harder classification scenario, where limited training data, taken from different persons than the validating ones exists. These results suggest that the proposed behavioural features provide an appropriate basis towards implementing an efficient real affective computing system. They can either be used to replace conventional, more obtrusive physiological measures, or in conjunction to them. In both cases, the results of the present study show that stress detection effectiveness can increase.

Moreover, considering future practical applications of automatic stress detection, it should be noted that the proposed features extracted from the video modality, form an unobtrusive activity-related behaviour monitoring framework that is based on a low-cost camera. Additionally, their extraction is based only on the silhouette of the subject depicted through the MHIs, together with the head's position. Both the MHIs and head position can be calculated in real-time. As a result, even for applying post-processing on the recorded data, the original video sequences that fully depict the subject are not required, and thus, these are not needed to be stored. Compared to typical facial expression recognition methods, it is thus clear that the proposed framework has higher chance for ethical acceptance in future practical applications.

Nevertheless, the accelerometer modality used in our study can not be regarded as unobtrusive as the video modality, and it can be considered rather obtrusive, similar to the typical physiological modalities. However, two comments should be made in this respect. First, our proposed framework is mainly based on video processing, since only one out of the seventy eight features examined was extracted from the accelerometers. Thus, the accelerometer modality could be omitted in a future practical system in order to make it as unobtrusive as possible, having a possible small degrade in performance. Second, different (e.g., vision-based) methodologies could be developed in the future so as to detect foot trembling, thus making the use of accelerometers unnecessary.

In the present analysis, behavioural features were extracted in off-line mode from video and accelerometer recordings, something that can also be done in practical clinical settings, where subjects can be monitored for a time period, and subsequently, behavioural features will be extracted, as soon as the monitoring period ends. However, considering further future practical applications that may be in need of real-time extraction of the proposed behavioural features, it has to be noted that by using multi-threading techniques, the proposed features can also be extracted in real-time, similarly to the procedure that should be followed for common physiological features. This way, our proposed parameters can provide further input also to a practical on-line stress detection system, so as to enhance its effectiveness.

Our analysis involved data collected during a situation that required sustained attention to a visual display. Such situations are typically addressed in various daily settings, including a typical day at the office, the monitoring of a safety critical system etc., where stress is highly likely to appear. Moreover, our behavioural features can also be extracted in further settings, which do not necessarily involve sustained attention over visual displays, however require for the subject to be sit in front of the monitoring camera. For instance, our proposed system could be applied at a psychologist's office, so as to monitor the patient's activity during the treatment session. It can be argued that physiological responses could be utilized for stress monitoring in further situations of diverse daily settings. However, where applicable, our proposed behavioural feature extraction system provides less obtrusive stress monitoring, which, as indicated from the results of our work, can significantly increase the effectiveness of conventional methods based on physiological responses. In any case, the proposed behavioural features can provide effective automatic stress detection in situations that constitute a rather fertile ground for the future application of automatic stress monitoring systems.

For instance, we came to realize that behavioural parameters can improve a stress detection system based on objectively measurable features, making it appropriate also for clinical settings.

Based on the idea that bodies are specific as word, some researchers began to speak of a “kinetic text,” defining the set of subjects' movements as “thick and specific as the words we speak” [54]. Our study emphasizes the role of automatic behavioural feature detection as a way, in conjunction to physiological measures, to objectifying the subjectivity.

*“Even patients lying on the couch move as they speak and convey their own rhythm and shape patterns. The patient's body position, movements of limbs, have an impact on the analyst whose movements, though perhaps unseen by the patient, are felt and heard so that both together form a kinetic, as well as verbal text.”* [54]

The sentence above highlights the importance to understand activity-related behavioural features also for a therapist. In a laboratory setting, such analyses can provide further information to the researcher for detecting situations hardly conveyable otherwise. To our knowledge this study represents one of the first attempts towards a system for activity-related automatic stress detection. We believe that the relevance of activity in the understanding of human behaviour is a cutting-edge and relevant theme in science and for future technological development. Our results are a preliminary step towards a complete and effective development, while more studies are needed in this direction.
